# Identification of Novel Autoantigen in the Synovial Fluid of Rheumatoid Arthritis Patients Using an Immunoproteomics Approach

**DOI:** 10.1371/journal.pone.0056246

**Published:** 2013-02-13

**Authors:** Sagarika Biswas, Saurabh Sharma, Ashish Saroha, D. S. Bhakuni, Rajesh Malhotra, Muzna Zahur, Michael Oellerich, Hasi R. Das, Abdul R. Asif

**Affiliations:** 1 Department of Genomics & Molecular Medicine, Institute of Genomics and Integrative Biology, New Delhi, India; 2 Department of Clinical Chemistry, University Medical Center Goettingen, Goettingen, Germany; 3 Department of Clinical Immunology and Rheumatology, Army Hospital (Research and Referral), New Delhi, India; 4 Department of Orthopaedic, All India Institute of Medical Sciences, New Delhi, India; 5 Department of Biochemistry and Molecular Biology, University of Gujrat, Gujrat, Pakistan; University of Leuven, Rega Institute, Belgium

## Abstract

Rheumatoid arthritis (RA) is a chronic, autoimmune and inflammatory joint disease with a poorly understood etiology. Despite widespread diagnostic use of anti-citrullinated protein antibodies and rheumatoid factor proteins there is a strong demand for novel serological biomarkers to improve the diagnosis this disease. The present study was aimed to identify novel autoantigens involved in rheumatoid arthritis (RA) pathogenesis through immune-proteomic strategy. Synovial fluid samples from clinically diagnosed RA patients were separated on two-dimensional gel electrophoresis (2-DE). Samples from patients with non-RA rheumatisms (osteoarthritis and trauma) were used as controls. Immunoreactive proteins were spotted by Western blotting followed by identification through Q-TOF mass spectrometer analysis. Forty Western blots were generated using plasma from ten individual RA patients and 33 reactive spots were identified, 20 from the high molecular weight (HMW) gel and 13 from the low molecular weight (LMW) gel. Among the 33 common immunogenic spots, 18 distinct autoantigens were identified, out of which 14 are novel proteins in this context. Expression analysis of five important proteins, vimentin, gelsolin, alpha 2 HS glycoprotein (AHSG), glial fibrillary acidic protein (GFAP), and α1B-glycoprotein (A1BG) by Western blot analysis using their specific antibodies revealed their higher expression in RA synovial fluid as compared to non-RA samples. Recombinantly expressed GFAP and A1BG protein were used to develop an in-house ELISA to quantify the amount of autoantibodies in the RA patients. RA patients revealed an increase in the expression of GFAP and A1BG in the plasma as compared to osteoarthritis patients. Therefore, GFAP and A1BG can be proposed as potential new autoantigens of diagnostic importance for RA subjects. Further characterization of these proteins in rheumatoid arthritis will be helpful in understanding the role of these proteins in the disease pathogenesis providing new diagnostic tool with better specificity and accurate detection of the disease.

## Introduction

During the last decade Rheumatoid arthritis (RA) has evolved rapidly, affecting about 0.5–1.0% of the general population. Etiology of the disease most likely involves genetic risk factors, activation of autoimmune response as well as environmental factors. The disease is systemic at all stages, characterized by inflammatory cell infiltration, synovial cell proliferation, destruction of cartilage and aberrant post-translational modifications of self-proteins that may play a role in breaking T and B cell tolerance. However, in patients with established disease, a synovial manifestation clearly dominates [1], [2].

The early clinical presentation may not be specific since RA is initially indistinguishable from other forms of arthritis. So far, there is no single biomarker for the early detection of RA. The characteristic feature of this disorder is the presence of autoantibodies in the patient serum that distinguishes it from non-autoimmune joint pathogenesis like reactive arthritis or osteoarthritis (OA) [3]. Among the immunologic detections, rheumatoid factor is the best-known autoantibody present, however, one third of RA patients have no rheumatoid factors. These antibodies are also reported in other disorders and even in up to 15% of the healthy population [4]. Currently, anti-citrullinated protein antibodies such as anti-filaggrin antibodies, anti-keratin and anti-Sa are used as serological markers for the early diagnosis of RA. But the overall sensitivity of all these anti-citrullinated protein antibodies has very little additional diagnostic value over rheumatoid factor alone [4]–[6].

Several other autoantibodies have been described in RA including antibodies against heat-shock proteins (Hsp65, Hsp90, DnaJ), immunoglobulin binding protein (BiP), heterogeneous nuclear RNPs, annexin V, calpastatin, type II collagen, glucose-6-phosphate isomerase (GPI), elongation factor human cartilage gp39 [7] and mannose binding lectin (MBL) [8]. There are some antigens such as citrullinated vimentin, type II collagen, fibrinogen and alpha enolase against which high titers of autoantibodies are specifically found in RA patients’ sera. Their levels are higher in synovial fluid than in serum [9], [10], but their presence in synovial fluid is less characterized and is not effective for the early detection [11]. More recent discoveries include antibodies to carbamylated antigens (anti-CarP), to peptidyl arginine deiminase type 4 (PAD4), to BRAF (v raf murine sarcoma viral oncogene homologue B1) and to 14 autoantigens identified by phage display technology [12].

The diagnosis of RA is still based on specific clinical parameters, radiographic evidence of joint destruction [13] and the presence of anti-CCP/rheumatoid factor antibodies/anti-MBL [14]. At present there is no specific test for monitoring disease progression and responsiveness to therapy. All the above assays including CCP assay do not reveal the information about the antigen specificity that initiate or perpetuate inflammatory autoimmune reactions in the joints [15], [16]. As a result diagnosis is critical and there is a strong need for novel and definitive serological biomarkers with higher sensitivity and specificity for an early diagnosis and prognosis of disease.

In this attempt to identify novel autoantigens and their respective antibodies in synovial fluid of clinically diagnosed RA patients, we used 2-DE followed by mass spectrometric analysis. Fourteen novel proteins were identified and out of them five were Western blotted using their specific antibodies. Recombinant proteins from two autoantigens were further analyzed using ELISA-based assay to demonstrate their utility and specificity for clinical diagnosis.

## Materials and Methods

### Sample Collection

#### Ethical statement

The study protocols were approved by medical ethics committee of Institute of Genomics and Integrative Biology, Delhi Department of Orthopaedic, All India Institute of Medical Sciences, New Delhi, India and Army Hospital (Research and Referral), Dhaula Kuwa, New Delhi, India. A written informed consent was obtained from all participating subjects.

Sample collection: A total of 131 Blood samples were collected in acid-citrate dextrose vacutainers from Indian population in the age range of 25–65 years. Out of these, 60 samples were taken from RA patients (35 females, 25 males); 30 from osteo arthritis (OA) petients (20 females, 10 males); 01 from trauma patient; and 40 healthy samples (20 female, 20 male) were collected as negative control. A detailed medical history of each patient was recorded. Clinical examinations including bilateral pain, swelling and erosion of small and big joints, early morning stiffness along with radiographs were obtained under the supervision of rheumatologists. Diagnosis of RA was based on established criteria by American College of Rheumatology/European league (Table 1), that include a health assessment questionnaire (HAQ) scoring, general health (GH), tender-joint count (TJC), swollen-joint count (SJC), and disease activity score (DAS) calculated from online DAS28 tool based on 28 joint count. The mean DAS score was 6.5 (range 4.5–7.0) and the patients with DAS score ≥5.0 were classified as severe in this study. The patients were being treated with disease-modifying anti-rheumatic drugs from 1–1.5 years. Synovial fluid samples were collected in chilled, sterilized culture tubes from clinically inflamed knee joints and stored at −80°C.

**Table 1:** Clinical and demographic characteristics of the study subjects.

S.N.	Characteristics	RA (N = 60)	Healthy control (N = 40)
1.	Age (years)	30–65	30–55
2.	Sex (M/F)	F = 35, M = 25	F = 20, M = 20
3.	Tender joints	14±5	2±2
4.	Mean ± SDswollen jointcount	15±3	2±2
5.	Duration of RA	5±3	0
6.	Serum CRP level (mg/liter)	80±20	10±5
7.	Multiple erosions	yes	No
8.	RF (IU/ml)	90±20	10±5
9.	Anti CCP (EU)	55±15	10±5
10.	ESR (mm/hr)	85±15	10±5
11.	DAS28	6±2	2±2

M-male, F-female, SD-standard deviation, CRP-C-reactive protein, mg-milligram, RF-rheumatoid factor, IU-international units, ml-milliliter, anti CCP- Anti-cyclic citrullinated peptide, EU- ELISA units, ESR-erythrocyte sedimentation rate, mm-millimeter, hr-hour, DAS28- disease activity score (28 joints maximum).

### Two-dimensional Gel Electrophoresis (2-DE)

For protein sample preparation, synovial fluids were centrifuged at 15000 rpm for 15 min at 4°C to remove blood contamination. The clear supernatants were digested with hyaluronidase enzyme (HSE) and purified according to the protocol given by Liao et al [9]. Total protein concentration was estimated by Bradford method [17]. Proteins (50 µg) were separated by 2-DE [18] in the first dimension by isoelectric focusing (IEF), using 3–10 immobilized pH gradient (IPG) strips (Bio-Rad, Munich, Germany) and in the second dimension by SDS-PAGE. IEF was carried out at 200 V for 1 h, 500 V for 1 h followed by ramping at 1000 V for 1 h and final focusing at 8000 V for a total of 13000 Vh. IPG strips were then equilibrated for 20 min in sodium dodecyl sulfate (SDS) equilibration buffer (50 mM Tris-HCl, pH 8.8, 6 M urea, 4% (w/v) SDS, 20% (w/v) glycerol) containing 2% (w/v) dithiothreitol and then for 20 min in the equilibration buffer containing 2.5% (w/v) iodoacetamide. After IEF, SDS-PAGE was performed in a Mini Protean II slab cell vertical system (Bio-Rad, Munich, Germany)**.** Gels were run for 8 h using 10% SDS-PAGE for high molecular weight (HMW) and for 2 h using 12% SDS-PAGE for low molecular weight (LMW) protein separation at 30 mA constant current followed by silver nitrate staining. Gels were scanned with a gel Cano scan 8400 (Canon, Tokyo, Japan).

### Western Blotting

The 2-DE gels were electroblotted overnight onto a nitrocellulose (NC) membrane (Amersham Biosciences) using Mini Protean wet transfer unit (Bio-Rad, Hercules, CA) at 50 V, 4°C [19]. The transfer of proteins onto the membrane was visualized by Ponceau staining followed by scanning. The membranes were blocked by incubation with 5% bovine serum albumin (BSA) followed by washing with TRIS-saline buffer containing 0.05% tween 20 (TBST). Blood plasma samples were diluted (1∶500) with 5% BSA in TBST and used as primary antibody. Membranes were incubated overnight with diluted primary antibodies at 4°C, washed with TBST followed by 2 h incubation at room temperature with diluted (1∶3000) anti-human IgG antibody conjugated with horseradish peroxidase (HRP) (Sigma Aldrich, Germany). The excess secondary antibody was removed by repeated washings with TBST before developing. The conjugate that remained bound on the membrane was detected by enhanced chemiluminescence assay (ECL) (Thermo Scientific, Rockford, IL) and autoradiographed on high performance chemiluminescence film (GE Healthcare) according to the manufacturés guidelines.

### In Gel Digestion of Proteins and Extraction of Peptides

Protein spots were excised in 1 mm cubes from the gels, washed with distilled water and destained with 1∶1 solution of 30 mM potassium ferrycyanide and 100 mM sodium thiosulphate. Dehydration of gel pieces were carried out with 1∶1 acetonitrile (ACN): water for 15 min and then with ACN till it became white and sticky. The gel pieces were then equilibrated with 100 mM ammonium bicarbonate and treated with ACN followed by drying in a vacuum centrifuge (UNIVAPO 150H, UniEquip, Martinsried, Germany). The gel pieces were then digested with trypsin (Promega, Germany) [20] . The peptides were extracted by varying the concentration of ACN and trifluoroacetic acid (TFA), sonicated, dried and stored in 0.1% formic acid at −20°C till further analysis.

### Q-TOF Analysis

Mass spectrometric analysis was carried out by using Micromas Q-TOF Ultima Global Mass Spectrometer (Waters, Manchester, UK) as described previously [20]. The reconstituted peptide samples (1 µL) were introduced to CapLC autosampler (Waters, Manchester, UK) onto-precolumn cartridge (C18 pepMap, 300 *í*m _ 5 mm; 5 *í*m particle size, LC Packings Idstein, Germany) and separated in an analytical column (C18 pepMap100 nano Series, 75 *í*m −15 cm; 3 *í*m particle size) LC Packings (Germany). The data were acquired on a Windows NT PC using MassLynx (v 4.0) software and data files were initially processed using ProteinLynx Global Server (PLGS, v 2.2, Waters, Manchester, Germany) module to generate *.pkl files under the settings; Electrospray, noise reduction 10%, centrioid 80% with minimum peak width 4 channel, Savitzky-Golay, MSMS, medium deisotoping with 3% threshold, no noise reduction and no smoothing. The peaklists were searched using the online MASCOT (http://www.matrixscience.com) algorithm against the Swiss-Prot 55.5 (389 046 sequences; 139 778 124 residues) and NCBInr protein database. The whole database was used to retrieve the data using the search parameters set as follows: enzyme trypsin; allowance of up to one missed cleavage peptide; mass tolerance (±0.5 Da) and MS/MS tolerance (±0.5 Da); modifications of cysteine carbamidomethylation and methionine oxidation when appropriate with auto hits allowed only significant hits to be reported. The identification of proteins were carried out on the basis of two or more peptides whose ions scores exceeded the threshold, P≤0.05, which indicated the 95% confidence level for these matched peptides. The proteins were accepted as identified if the threshold was exceeded and the protein spot possessed the correct molecular mass and pI value in the corresponding gels. All the experiments were repeated at least thrice, protein spots were digested from more than one gel and analyzed with mass spectrometry to ensure accurate identification.

### Western Blotting for Auto-antigens Verification

Confirmatory Western blot analysis was carried out as described above. The RA and OA patients’ synovial fluid (50 µg protein) samples were separated on 12% SDS gel, proteins were electro-transferred to the NC membrane and NC membrane was incubated with the five different primary antibodies*:* vimentin, gelsolin, AHSG, GFAP and A1BG at 1∶8000, 1∶5000, 1∶10,000, 1∶8,000 and 1∶8,000 dilution respectively. Mean density of blots were calculated by alpha DG Doc software followed by statistical analysis using Students two-tail t-test. The results were expressed as mean ± SD and p≤0.01 was considered as statistically significant value. The immuno-reactive protein spots obtained after Western blotting were matched using Delta2D v 4.0 (Decodon, Germany) as well as manually with the corresponding silver stained 2-DE gels.

Presence of autoantibodies against GFAP and A1BG were confirmed after separating 0.5 µg of GFAP and A1BG recombinant pure protein on 12% SDS gel followed by Western blot with diluted (1∶200) plasma samples of RA, OA and healthy patients as source of primary antibodies. Anti-human IgG HRP conjugated having dilution 1∶20,000 was used as secondary antibody and developed by ECL as mentioned in Western blotting section.

### Quantification of Auto-antibodies by ELISA

Levels of autoantibodies against GFAP and A1BG were measured in the plasma of RA patients (n = 30), OA (n = 30) and healthy individual (n = 30) by ELISA as described previously (17) with slight modification. Each well of a 96 well microtiter plate was coated in triplicate with recombinant pure protein having 0.20 µg/100 µl concentrations (0.01 M Na_2_CO_3_ and 0.035 M NaHCO_3_, pH 9.6). The plates were left at 4°C for 24 h, washed three times with 100 µl TBST and incubated with 100 µl blocking buffer (1% BSA in TBS) for 1 h at room temperature. The blocking buffer was removed, washed and incubated with 100 µl of RA, OA and healthy patients’ diluted plasma (1∶200 dilution) separately as primary antibodies for 3 h at RT. The plates were then washed (three times) as before and incubated with 100 µl of anti-human HRP conjugate for 1 h and developed with ortho phenylene diamine (1 mg/ml in 0.05 M citrate buffer and 5 µl/ml H_2_O_2_) for 30 min. The reaction was stopped by adding 2 N H_2_SO_4_ to each well and binding efficiency was checked by reading absorbance at 492 nm in an ELISA reader (Spectramax Plus; Molecular Devices, USA).

The expression (concentration) of antigens (GFAP and A1BG proteins) in the synovial fluids was measurement by coating synovial fluid (diluted 1∶200) of RA (n = 30), OA (n = 30) in triplicate in 96 well microtiter plate for 2 h at RT. Glial fibrillary acidic protein (GFAP) and A1BG at 1∶2000 and 1∶2500 dilution respectively were used as primary antibodies separately and anti-mouse HRP conjugate was used as secondary antibody. The antigens (GFAP and A1BG proteins) expression was estimated by reading absorbance at 492 nm in an ELISA reader as described above.

## Results

### Autoantigens from Synovial Fluid of RA Patients

Clinical demographic studies and DAS score calculations were applied to plasma and synovial fluid samples of 60 RA patients as well as 40 healthy controls (Table 1). To detect autoantigens, synovial fluid proteins from 10 RA patients were separated by 2-DE and transferred to NC membrane. Each membrane was treated with one plasma sample as primary antibody and with secondary antibody as mentioned above. Western blots were categorized according to molecular weights of antigens. Antigens having molecular weight less than 75 kDa were designated as low molecular weight (LMW) and more than 75 kDa as high molecular weight (HMW) antigens. A total of 40 Western blots; 10 Western blots of HMW antigens and 30 Western blots of LMW antigens were generated, documented and further visualized by silver staining for identification purpose (Figure S1). Plasma from patients with rheumatoid arthritis reacted with synovial fluid in 33 common immunogenic spots: 20 were HMW and 13 were LMW spots (Figure S2 B, S2D) while a far less number of spots were given by Western blots of OA synovial fluids (Figure S2A, S2C). The negligible reactivity was observed in control Western blots of trauma patients’ synovial fluid (Figure S3).

### Identification of Reactive Proteins

The reactive spots (shown in Figure S2B and S2D) were excised from corresponding silver stained gels and the peptides were extracted from excised gel after in-gel enzymatic digestion and analyzed using Q-TOF mass spectrometry. Out of 33 immunogenic spots, 18 different proteins were identified by mass spectrometric analysis (Table 2). Some proteins were identified in more than one spots in both HMW and LMW region. These are: fibrinogen (spot no. 1, 22, 23), plasma protease C1 inhibitor (spot no. 3, 31), alpha 1 antitrypsin (spot no. 2b, 16, 18, 30) and vimentin (spot no. 10, 26). Other HMW antigens identified in single spot are: gelsolin (spot no. 4), A1BG (spot no. 5, a representative MS/MS spectra shown in Figure S5) and GFAP (spot no. 9, a representative MS/MS spectra shown in Figure S4). In LMW region, inter alpha trypsin inhibitor (spot no., 2a and 6a ), ceruloplasmin (spot no. 6, 7 and 15), complement factor H (spot no. 8, 12), alpha 2 macroglobulin (spot no. 11, 13, 14 and 27), serum amyloid (spot no. 19), complement C3 (spot no. 27, 28, 29), C-reactive protein (spot no. 20), serum albumin (spot no. 21), serotransferrin (spot no. 24) and AHSG (spot no. 25) were identified. Nine spots were identified as IgG kappa chain and lambda chain in HMW, whereas IgG gamma chain was identified in both HMW and LMW regions in seven spots (not shown). The observed molecular weight of each spot was determined by DG Doc 1201 software.

**Table 2:** Identified high and low molecular weight RA antigens.

Spot No.	Accession No.	MW(kDa)Obs/Thr^*^	pI Obs/Thr^#^	MOWSEScore	Peptides Matched	Protein name
		High molecular weight (HMW) antigens				
1	P02679	110.00**/**51.47	5.8**/**5.3	323	11	Fibrinogen gamma chain
2a	P19827	100.00**/**101.3	5.4**/**6.3	149	5	Inter-alpha-trypsin inhibitor heavy chain H1
2b	Q86U18	98.00**/**46.70	5.2**/**5.3	173	4	Alpha -1- antitrypsin
3	Q96FE0	90.00**/**55.11	4.8**/**6.0	170	4	Plasma protease C1 inhibitor
4	P06396	80.00**/**85.64	7.2**/**5.9	73	3	Gelsolin
5	P04217	82.00**/**54.23	5.4**/**5.5	156	4	Alpha 1-B glycoprotein
6	P00450	140.00**/**122.1	5.8**/**5.4	387	12	Ceruloplasmin
6a	Q14624	125.00**/**103.2	6.0**/**6.5	46	5	Inter-alpha-trypsin inhibitor heavy chain H4
7	P00450	145.00**/**122.1	6.5**/**5.4	491	18	Ceruloplasmin
8	Q9NU86	170.00**/**139.0	6.2**/**6.2	108	7	Complement factor H
9	Q9UFD0	200.00**/**49.85	4.5**/**5.4	70	3	Glial fibrillary acidic protein
10	P24789	250.00**/**52.81	4.2**/**5.1	78	3	Vimentin
11	P01023	190.00**/**163.1	4.0**/**6.0	367	14	Alpha 2 macroglobulin
12	Q9NU86	170.00**/**139.0	6.4**/**6.2	437	17	Complement factor H
13	P01023	165.00**/**163.1	6.8**/**6.0	404	14	Alpha 2 macroglobulin
14	P01023	170.00**/**163.1	6.8**/**6.0	154	9	Alpha 2 macroglobulin
15	P00450	120.00**/**122.1	5.4**/**5.4	185	7	Ceruloplasmin
16	Q86U18	120.00**/**46.70	5.4**/**5.3	67	3	Alpha-1-antitrypsin
17	P01023	165.00**/**163.1	6.0**/**5.2	50	5	Alpha 2 macroglobulin
18	Q86U18	205.00**/**46.70	5.3**/**6.1	75	4	Alpha-1-antitrypsin
		Low molecular weight (LMW) antigens				
19	P02743	23.05**/**25.37	6.2**/**6.1	114	4	Serum amyloid
20	P02741	22.87**/**25.02	5.8**/**5.4	53	3	C-reactive protein
21	Q9P157	92.76**/**69.32	6.5**/**5.9	1402	117	Serum albumin
22	P02675	44.50**/**55.89	6.5**/**8.5	331	6	Fibrinogen beta chain
23	P02675	44.84**/**55.89	6.2**/**8.5	185	8	Fibrinogen beta chain
24	P02787	99.37**/**77.00	7.5**/**6.8	1885	83	Serotransferin
25	P02765	72.62**/**39.30	5.2**/**5.4	139	2	Alpha 2 HS glycoprotein
26	P24789	187.53**/**52.81	5.2**/**5.1	45	2	Vimentin
27	P01024	87.25**/**187.03	8.4**/**6.0	419	13	Complement C3
28	P01024	90.66**/**187.03	8.0**/**6.0	308	4	Complement C3
29	P01024	90.66**/**187.03	7.8**/**6.0	248	9	Complement C3
30	Q86U18	69.89**/**46.70	5.8**/**5.3	550	10	Alpha-1-antitrypsin
31	Q96FE0	114.92**/**55.11	4.5**/**6.0	125	4	Plasma protease C1 inhibitor

Identification of high molecular weight (HMW) and low molecular weight (LMW) antigens using Q-TOF mass spectrometric (MS) analysis followed by online MASCOT search against the SwissProt and NCBInr protein databases (kDa-kilo Dalton, MW-molecular weight, Obs-observed, Thr- theoretical, pI- isoelectric point, MOWSE- Molecular Weight Search).

**Figure 1:**
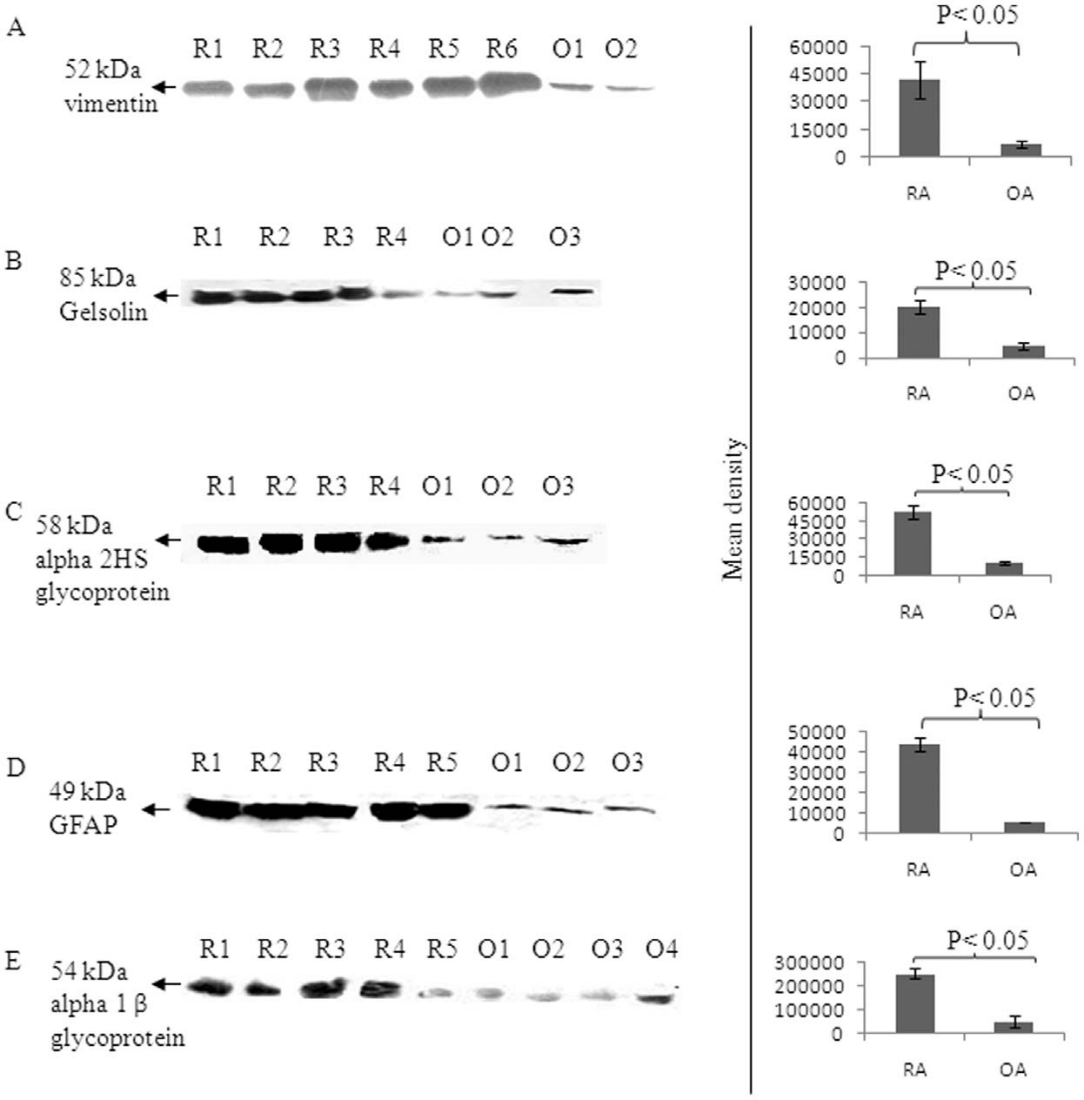
Expression analysis of vimentin, gelsolin, AHSG, GFAP and A1BG autoantigens by Western blotting. Synovial fluid of various RA (R1–R6) and OA (O1–O4) patients were separated on SDS-PAGE and Western blotted with (A) anti-vimentin, (B) anti-gelsolin, (C) anti-alpha 2HS glycoprotein, (D) anti-glial fibrillary acidic protein E) anti-alpha 1-B glycol protein antibody. The densitometry analysis is shown as bar diagram (right panels) and the OA patientś synovial fluids served as control sample.

Vimentin, gelsolin, AHSG, GFAP and A1BG antigens were further confirmed by Western blot analysis using their corresponding specific antibodies followed by densitometric analysis (Figure 1). The expression levels of vimentin, gelsolin, AHSG, GFAP, and A1BG were observed to be increased by 7, 5, 5, 8 and 5 fold respectively in the synovial fluid of RA patients as compared to synovial fluid of OA patients (Figure 1A–1E).

### GFAP and A1BG as Novel Auto Antigens

**Figure 2:**
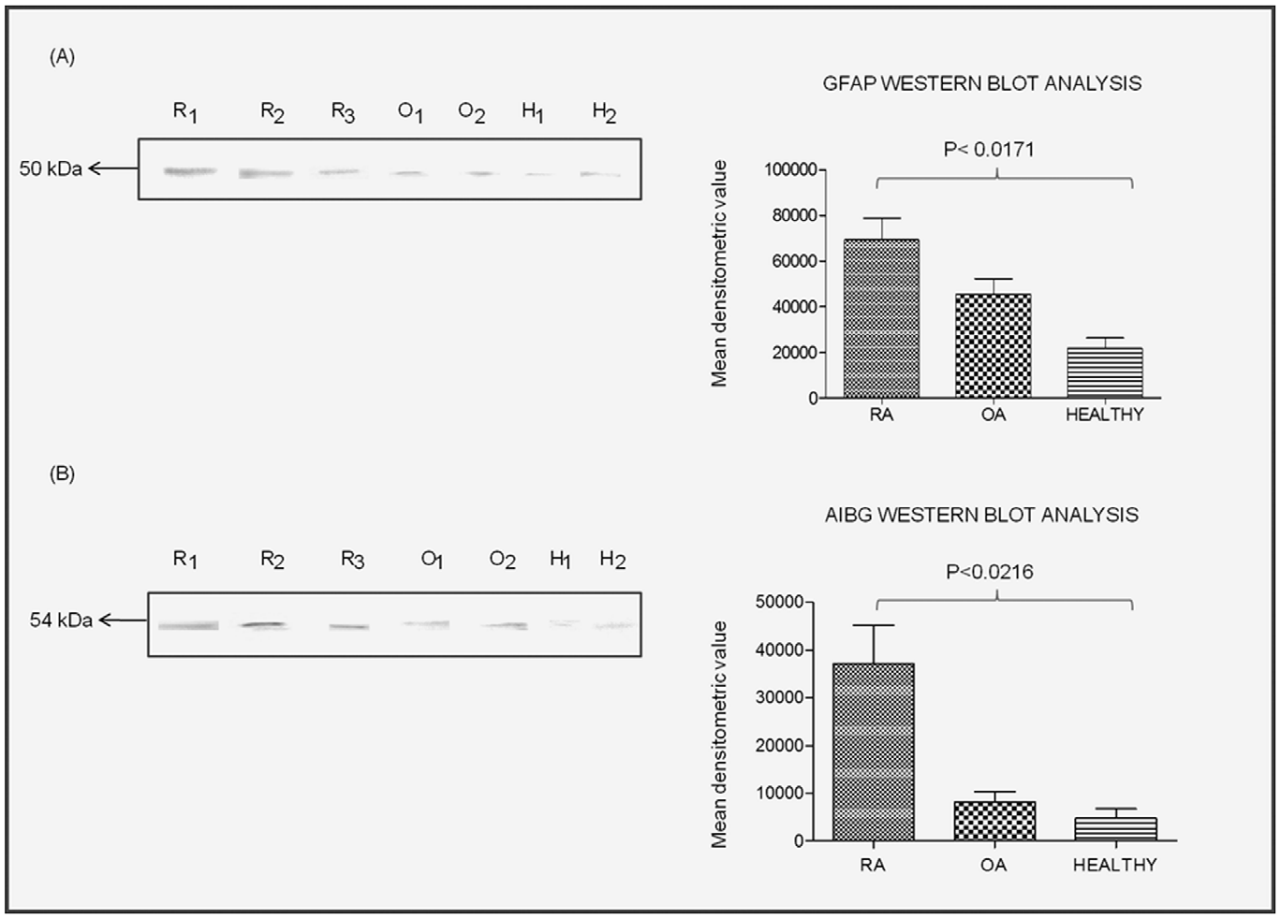
Detection of autoantibodies against purified GFAP and A1BG recombinant proteins in RA patients’ plasma. The purified GFAP and A1BG recombinant proteins were separated on SDS-PAGE and Western blotted (A) with RA patients’ plasma. Bar diagrams (B) shows the densitometry analysis results and bar represents the mean ± SD of the mean of each spot. Abbreviations: R1, R2 and R3 represent plasma of various RA patients; O1, O2 represents plasma of various OA patents’, H1, H2 represents plasma of various healthy controls.

Presence of autoantibodies against GFAP and A1BG in the plasma samples of RA patients were confirmed by Western blot analysis after separating the recombinant pure GFAP and A1BG proteins on SDS-PAGE. Differential reactivity of GFAP and A1BG auto-antibodies in different plasma samples of RA, OA and healthy controls were calculated by densitometric analysis (Figure 2A–2B, bar shown on the right side of Western blot results). The reactivity levels of GFAP was higher in RA plasma by 1.5 fold as compared to OA plasma samples while 3.5 fold than healthy control samples (Figure 2A). However, reactivity of A1BG in RA plasma was 5 fold higher than OA plasma and 6 fold than healthy control (Figure 2B).

**Figure 3:**
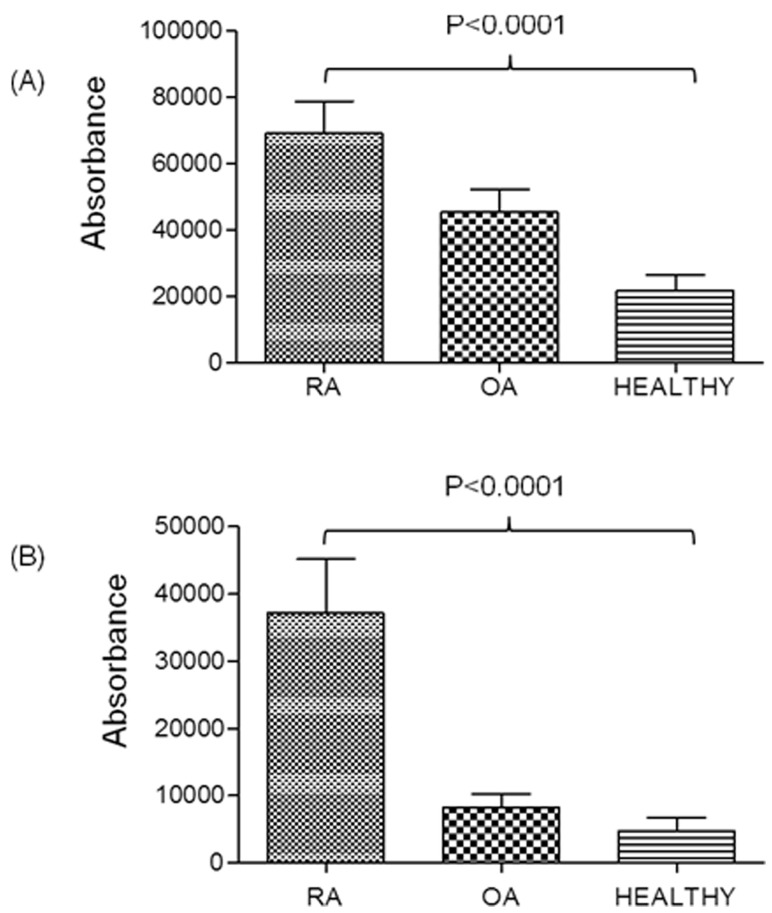
Quantification of autoantibodies in RA patient’s plasma by ELISA using recombinant pure protein of GFAP and A1BG. Confirmation of over-expressed (A) GFAP and (B) A1BG in plasma of RA patients (n = 30) compared to OA (n = 30) and healthy controls (n = 30). Densitometric analysis showed higher expression level of both proteins in the plasma of RA patients compared to OA and healthy controls.

The expression levels of autoantibodies against GFAP and A1BG were recorded in large number of plasma samples (samples were different than used for 2-DE and Western blotting) of RA, OA and healthy control (n = 30 each), by coating ELISA plate with recombinant pure proteins of GFAP and A1BG. It was observed that autoantibodies against GFAP is expressed 1.4 fold and 3.5 fold higher in RA plasma compared to OA and healthy control (Figure 3A) whereas autoantibodies against A1BG is expressed 4 fold and 5 fold more in RA than OA plasma and healthy control respectively (Figure 3B).

**Figure 4:**
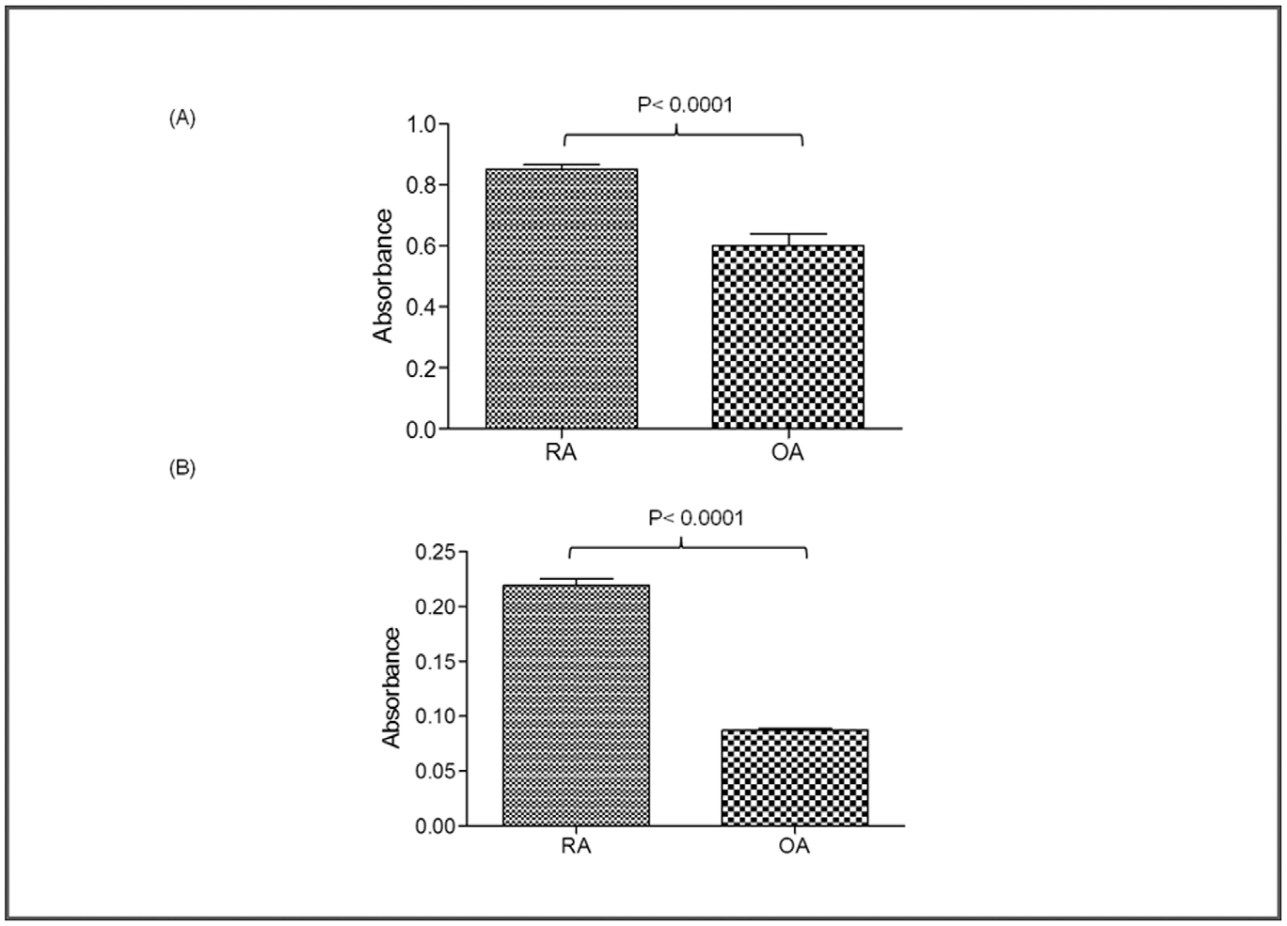
Analysis of increased expression of GFAP and A1BG autoantigens in the synovial fluids of rheumatoid arthritis patients by ELISA. Increased expression of (A) GFAP (B) A1BG in rheumatoid arthritis compared to osteoarthritis synovial fluid samples.

Likewise, ELISA was carried out by coating various synovial fluid samples of RA (n = 30) and OA (n = 30) incubated with antibodies against GFAP and A1BG. The results showed that the expression level of GFAP and A1BG was 1.5 fold (Figure 4A) and 3 fold higher in RA as compared to OA patients respectively (Figure 4B).

## Discussion

The identification of disease-specific autoantigens is essential for understanding the pathogenesis of autoimmune diseases and defining markers for detection of preclinical autoimmune disorders [21]. It is unquestionable that novel biomarkers are required for a better diagnosis of early and seronegative RA. Early treatment in RA is important as it can prevent irreversible damage of the joints. Among the 18 reported autoanigens in this study, 14 autoantigens were first time identified in synovial fluid of RA patients. Four antigenic proteins: Fibrinogen, vimentin, C-reactive protein (CRP) and IgG have already been reported in this disease. Vimentin and fibrinogen were reported as citrullinated and predominantly associated with RA patients [22]–[24]. Further, citrullinated vimentin has also been identified as a native antigen for anti-Sa antibodies [6] and citrullinated fibrinogen (α and β chains of fibrin) was reported to be a candidate autoantigen of anti-citrullinated protein antibodies that were frequently detected in RA synovial fluid [5], [22]. In non-erosive RA plasma, CRP, an acute phase protein, was increased 2–12 fold higher and 47–142 fold higher in erosive RA plasma than that in healthy control [9]. Serum amyloid is another acute phase protein like CRP reported in RA. It was observed that serum amyloid A is even more sensitive than CRP in response to the inflammatory lesion of rheumatoid arthritis [25].

Out of 14 new autoantigens identified, we examined the expression level of 4 new autoantigen proteins (gelsolin, AHSG, GFAP and A1BG) and one known autoantigen (vimentine) in RA synovial fluid. Gelsolin is an intracellular actin-binding protein involved in cell shape changes, cell motility, and apoptosis and is the only known Ca^2+^dependent severing protein identified [26]. It has been suggested to be a key component of an extracellular actin-scavenging system during tissue damage, making it a protein of interest to study in RA scenario. The expression level of gelsolin was found to be increased by 5 folds in RA synovial fluid than control (Figure 1B) while in an another report plasma gelsolin level was found to decrease in RA patients [27].

The serum AHSG level has been reported to be decreased significantly in RA patients as well as patients suffering from diabetes mellitus, malnutrition and cardiovascular disease [28]. But in this study, AHSG showed 5 fold increased expression in RA synovial fluid than control (Figure 2C), however, its role in RA is still not clear. Likewise GFAP protein was observed to be increased in RA patients in this report than OA and healthy control (Figure 1D, Figure 2, 3, 4). GFAP is expressed in astrocytes, ependymal cells and other cell types in central nervous system and is closely related to vimentin, desmin and peripherin that are linked to inflammation. It helps to maintain astrocyte mechanical strength and the shape of the cells but its exact function is poorly understood. The phosphorylation/dephosphorylation of GFAP is important for the modulation of its assembly/disassembly cycle [29]. Further, the structural plasticity of glial filaments and the functional activities of astrocytes were found to be regulated by the phosphorylation of GFAP along with vimentin, a ubiquitous mesenchymal intermediate filament [30]. Vimentin association with RA is well reported [23] and since GFAP is closely related to vimentin, we choose GFAP for further confirmatory experimentation.

α1B-glycoprotein (A1BG) also known as the binding partner of Cysteine-Rich Secretory Protein 3 (CRISP-3) is known to be present in exocrine secretions and in secretory granules of neutrophilic granulocytes. It is believed to play a role in innate immunity thus activate the adaptive immune system through a process known as antigen presentation [31]. Immunological role should make it a protein of interest to study in autoimmune diseases like RA. A1BG is present in very low concentration in human plasma and amniotic fluid [32]. We observed 5 fold higher expression of A1BG in RA than in control synovial fluid (Figure 1E) which was also confirmed by Western blot analysis and ELISA (Figure 2, 3, 4). This is the first report where we found the presence of GFAP and A1BG in RA synovial fluid so far. The Western blot analysis and ELISA results more specifically confirmed the presence of GFAP as well as AIBG autoantibodies in RA plasma. Among the other new autoantigens, ceruloplasmin (Cp) was reported to be present in the range of 0.2–0.5 mg/mL in the normal human serum whereas it was observed to be increased by 2 fold in RA patient sera [33]. Alpha 2 macroglobulin was found to degrade cartilage matrix in RA when complexed with elastase in synovial fluid of RA patients [34]. Factor H was found to be present in synovial fluid of RA patients and is produced locally at the site of inflammation. It acts as protective molecules to reduce C3b deposition on the surface of host cells and may participate in local defense reactions during pathologic processes [35]. Inter alpha trypsin inhibitor (ITIH) and plasma protease C1 inhibitor antigen were found to be unaltered in RA patients and the role of complement C3 is not clear in RA. But C3 activation products were reported to be present in RA plasma [36]. The in-depth study of the above proteins may lead us to understand the mechanism of pathogenesis of RA disease.

In conclusion, here we report several new as well as previously reported autoantigens of RA. This is the first report in which GFAP and A1BG autoantigens are shown to be associated with RA and autoantibodies against GFAP and A1BG were isolated in RA plasma. Hence GFAP and AIBG may be used as biomarkers for RA pathogenesis along with other clinical parameters. Thus, our immunoproteomics approach for autoantibody profiling of RA autoimmune diseases is a convenient and effective tool for detecting autoantigens. It may also serve as a tool for predictive screening for the identification of novel autoantigens in other autoimmune diseases.

## Supporting Information

Figure S1 **2-DE from synovial fluid proteins of RA patients.** (A) Silver stained pattern of synovial fluid proteins of RA patients in HMW (75–200 kDa) regions and (B) in LMW (25–200 kDa) regions. The arrows indicate the protein spots analyzed by MS/MS analysis.
(TIF)

Figure S2 **Detecting of antigens of RA autoantibodies’ by Western blot analysis.** (A &, C) Immunogenic spots obtained after Western blotting of OA patients synovial fluid with plasma in HMW and LMW regions respectively. (B & D) Immunogenic spots obtained after Western blotting of RA patients synovial fluid with their plasma in HMW and LMW regions respectively.
(TIF)

Figure S3 **Immunogenic spots in synovial fluid of healthy control (trauma patient).** Immunogenic spots from synovial fluid of control (trauma patient) sample in A) HMW and B) LMW region were generated using plasma as primary antibodies.
(TIF)

Figure S4 **Mass spectrometric analysis.** MS/MS analysis of GFAP using Q-TOF mass spectrometer.
(TIF)

Figure S5 **Mass spectrometric analysis**. MS/MS analysis of A1BG using Q-TOF mass spectrometer.
(TIF)
