# Supplementary figures and images for: Identification of Novel Autoantigen in the Synovial Fluid of Rheumatoid Arthritis Patients Using an Immunoproteomics Approach
Source: PLoS One. 2013 Feb 13;8(2):e56246. doi: 10.1371/journal.pone.0056246 (PMC3572018; doi:10.1371/journal.pone.0056246)

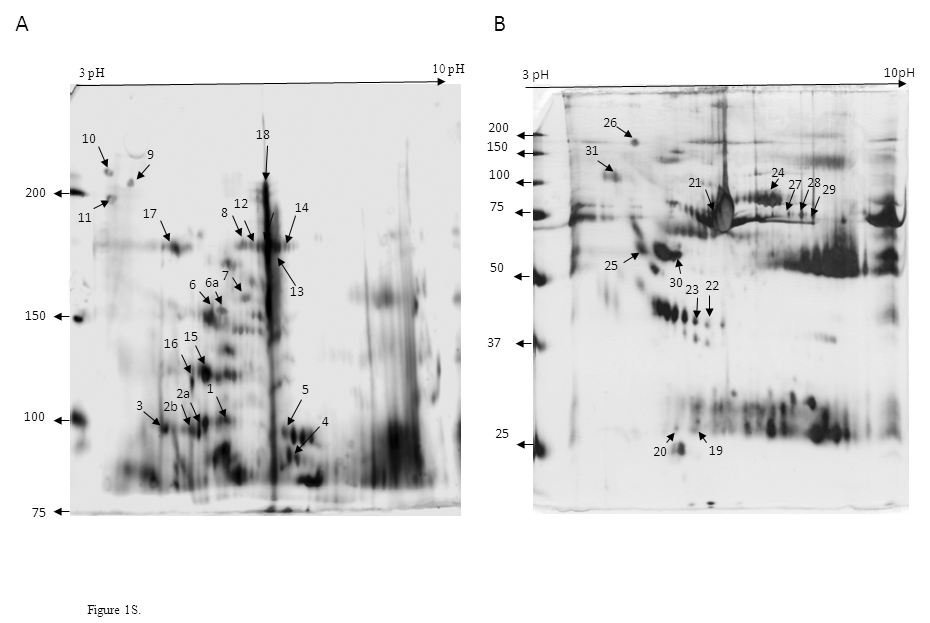

Supplement: Figure S1 — 2-DE from synovial fluid proteins of RA patients. (A) Silver stained pattern of synovial fluid proteins of RA patients in HMW (75–200 kDa) regions and (B) in LMW (25–200 kDa) regions. The arrows indicate the protein spots analyzed by MS/MS analysis. (TIF) [file pone.0056246.s001.tif]

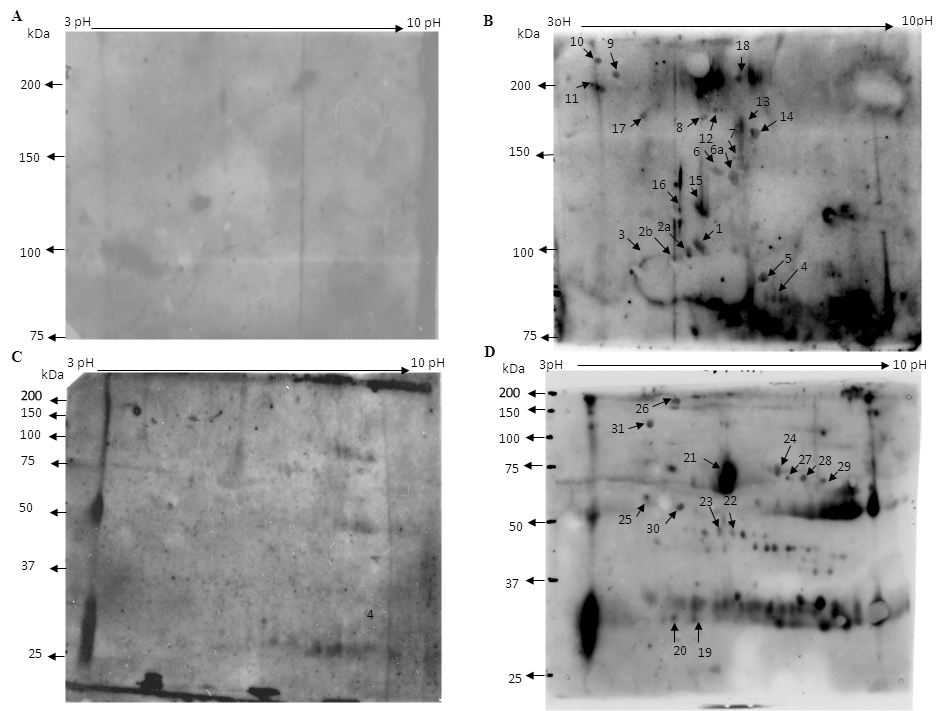

Supplement: Figure S2 — Detecting of antigens of RA autoantibodies’ by Western blot analysis. (A &, C) Immunogenic spots obtained after Western blotting of OA patients synovial fluid with plasma in HMW and LMW regions respectively. (B & D) Immunogenic spots obtained after Western blotting of RA patients synovial fluid with their plasma in HMW and LMW regions respectively. (TIF) [file pone.0056246.s002.tif]

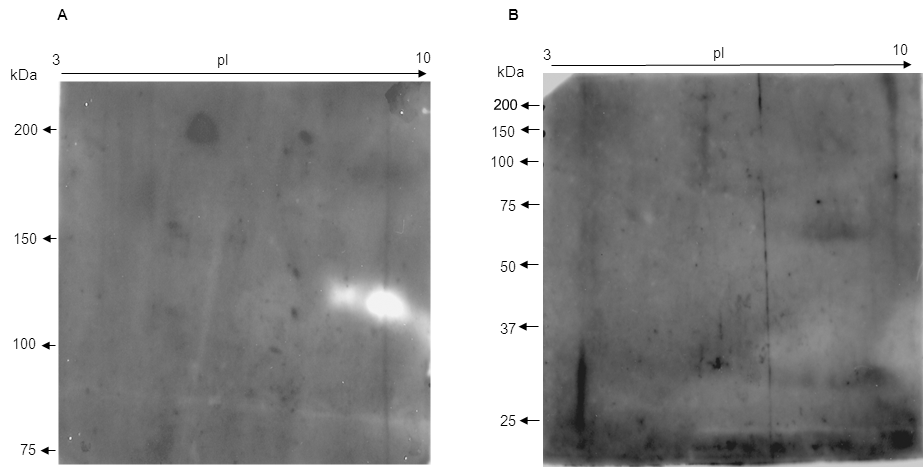

Supplement: Figure S3 — Immunogenic spots in synovial fluid of healthy control (trauma patient). Immunogenic spots from synovial fluid of control (trauma patient) sample in A) HMW and B) LMW region were generated using plasma as primary antibodies. (TIF) [file pone.0056246.s003.tif]

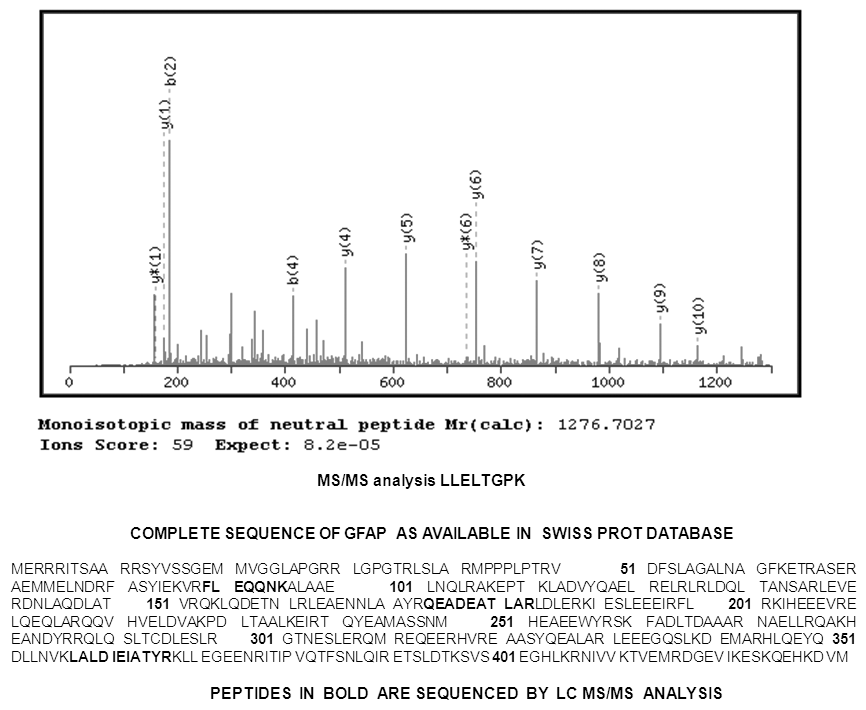

Supplement: Figure S4 — Mass spectrometric analysis. MS/MS analysis of GFAP using Q-TOF mass spectrometer. (TIF) [file pone.0056246.s004.tif]

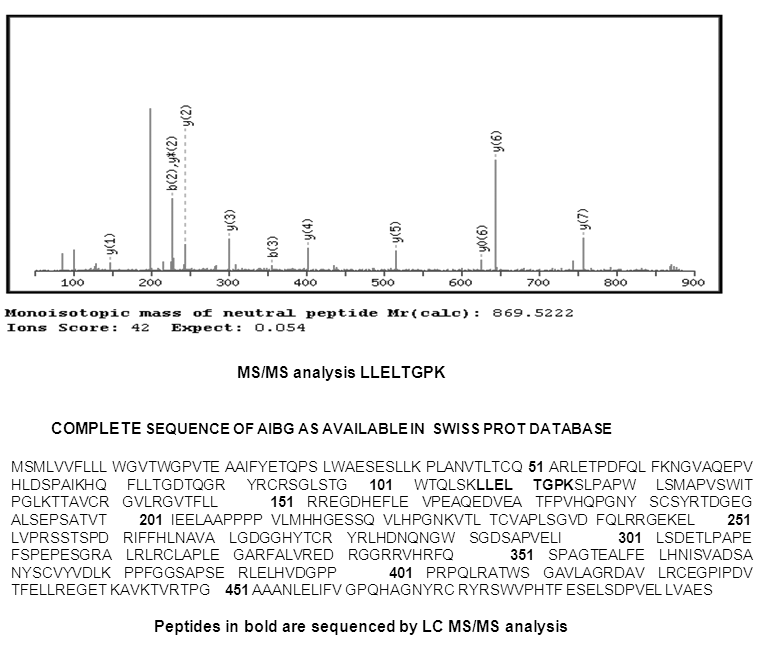

Supplement: Figure S5 — Mass spectrometric analysis. MS/MS analysis of A1BG using Q-TOF mass spectrometer. (TIF) [file pone.0056246.s005.tif]
